# Remarks on Changes of Structure in Organised Bodies

**Published:** 1828-08

**Authors:** John Baron


### THE LONDON
### Medical and Physical Journal.

N° 354, VOL. LX.] AUGUST, 1828. [N° 26, *New Series*.

For many fortunate discoveries in medicine, and for the detection of numerous errors, the world is indebted to the rapid circulation of Monthly Journals; and there never existed any work, to which the Faculty, in Europe and America, were under deeper obligations, than to the *Medical and Physical Journal of London*, now forming a long, but an invaluable, series.—RUSH.

### ORIGINAL PAPERS,

AND

### CASES OBTAINED FROM PUBLIC INSTITUTIONS AND OTHER AUTHENTIC SOURCES.

### CHANGES OF STRUCTURE.

*Remarks on Changes of Structure in Organised Bodies.*
By JOHN BARON, M.D. F.R.S. &c. &c.

As the pathological investigations in which I have been for many years engaged are again occupying some portion of the attention of medical inquirers, I am desirous, if possible, to remove some misconceptions and prejudices which appear to me to stand in the way, and to impede the progress of knowledge. On that account I have taken the liberty of submitting to you a few remarks, which I trust will receive a place in your valuable Journal.

Nothing, perhaps, has tended more to keep the minds of professional men in a state of doubt and hesitation with regard to the views I have attempted to unfold, than the circumstances under which they are constrained to pursue their examinations concerning changes of structure in the human body. I would, therefore, earnestly solicit them to consider the matters of fact I am about to submit to them. In human pathology, except on very rare occasions, we never witness disorganizations until they have arrived at their last and fatal change. We can seldom examine them at their commencement; can rarely trace their progress; and are compelled, therefore, to draw inferences from appearances which can throw but little light upon the subject. It is my conviction that this is the cause why we have gained so little satisfactory information regarding the origin and course of morbid changes. It seems equally certain that so long as we confine ourselves to human pathology *alone*, this difficulty must continue. In order to overcome it, I have endeavoured to follow a plan somewhat different from what has generally been adopted. One of the conclusions which has been come to is, that the primary condition of many of the most important alterations of texture is so different from the ultimate one, that it is impossible to draw any just inference as to the former from what may be observed in the latter. I feel quite assured that inattention to those points has caused many statements, that are perfectly well grounded, to be rejected as conjectural or untrue. It is on that account especially to be desired that inquirers in this field should divest themselves of this prejudice, and not refuse to admit descriptions of incipient disorganization, because they do not find them realised when examination is made in the more advanced stages.

It is, nevertheless, certain that, in many disorganizations, particularly those of a compound nature, we find indications by which the different steps in their progress may occasionally be detected: thus, in one part of a viscus we may detect the disorder just beginning to form; in another, it may be somewhat more advanced; and in a third, it shall have proceeded still farther. The same conclusion may often be drawn from examining various organs in the same diseased body. One organ may be a little changed; another may be entirely altered.

Observations of this kind led me to believe it possible to trace these alterations in structure with much accuracy. The most important matter was to ascertain the earliest deviation from the healthy state. This could only be done, in the human subject, in those cases where an individual had been cut off whilst the disorganising process was going on. That point being ascertained, the inferences deducible from the indications just referred to became of much moment. It demonstrated a connexion between things apparently very dissimilar, and gave reason for believing that difference in appearance did not denote so much actual difference in nature as in progress. It is manifest that, from examination of the human body alone, all the steps in this progress could not well be made out. Luckily, in this difficulty, there was a resource to be found in the organic changes of the inferior animals. The analogy between the structure of their bodies and that of man is sufficiently close to permit us to avail ourselves of such information as may be derived from tracing disease in them, in order to elucidate our imperfect observations in the human subject. To this extent, and no further, did I think it advisable to proceed in such an inquiry. In no instance have I deduced any conclusions from examining the diseases of the inferior animals alone. I have looked upon comparative pathology merely as a help to human pathology; and a most important one it is. The slight and insufficient proofs of the course of disease which the latter affords may often be most happily and satisfactorily sustained by the latter.

I could have wished that these considerations had been borne in mind by those who have animadverted on my opinions. This wish especially applies to a writer in the last Number of the Edinburgh Medical and Surgical Journal. He has, I think, misapprehended my meaning; and, inadvertently I would hope, not accurately represented my sentiments; and, were I to remain silent, it might be inferred that I acquiesced in his representation. With your permission, therefore, I will show wherein I have been misunderstood; and this, chiefly, because truth may thereby be impeded. The objections, moreover, which he has urged, I believe, are from the causes above explained, not uncommon with our brethren; and it is natural and right to remove them.

If I understand the drift of the reviewer's remarks, they lead to these inferences: First, that the facts I have adduced do not apply to the history of pulmonary tubercles in human subjects; that, in short, I have taken for granted the thing to be proved;—and, secondly, as if the whole of the doctrines which I have endeavoured to unfold respecting the origin, progress, and character of a great variety of disorganizations rested upon the same false foundation, the reader is led to believe that assumption, bare unsupported assumption, has been deluding me for the best part of my professional life. I mean briefly to examine these two propositions; and, for various reasons, I shall begin with the last.

Before entering into detail, I claim the privilege of disengaging the pathological question from all the difficulties which have arisen from the ambiguous meanings attached to the word *hydatid.* I foresaw these difficulties in my first work, and I did all in my power to guard against them. Among other expressions, I use the following: "The injudicious adoption of terms is a fertile source of error. It has frequently thrown difficulties in the way, by establishing inaccurate impressions, which, trifling as they may appear, may require a fortunate combination of circumstances and the strongest efforts of the mind to remove. This I conceive in some degree to have been the case with respect to the word hydatid. The etymology of that word, and the ideas which are naturally associated with it, have restricted *its* meaning, and *our* inquiries too; insomuch that we have scarcely ever meditated on these bodies in any other character but in that which their name designates. The power of that name seems almost to have prevailed in opposition to the testimony of our senses. For, though the transformations of hydatids had been seen going on by different observers,—though the same tumor had been found to contain all the gradations of substances, from the simple watery vesicle up to the scirrhous or cartilaginous texture,—though Boerhaave and others had referred distinctly to these transformations,—though Dr. John Hunter had supposed that they might occur, and Dr. Jenner had proved that they actually did occur, and thereby form tubercles in the lungs,—it is remarkable that such facts have neither influenced the reasonings nor the opinions of professional men." To show how very important I considered this view of the subject, and how completely I had anticipated the objections, I remarked in a preceding page, that, "admitting, for the sake of argument, that hydatids are animalcules, it has, I trust, been shown that it is to the loss of the hydatidical character altogether, and the transformations of these bodies, that the morbid appearances in this and many other diseases are to be referred. This is the broad ground upon which I wish to rest my view of the subject, My design has been to prove that such transformations do take place, and occasion a great variety of disorganizations in the animal frame; and I consider that the question, so far as pathology is concerned, has nothing to do with the speculations respecting the origin and vitality of hydatids."*

I have carried these same principles with me throughout all I have written since; and by them I wish to be judged. Wherever I have spoken of the hydatid, my design has been to consider it merely as "a vesicular body with fluid contents" formed in the animal frame, and occasioning, by its growth and transformations, an immense variety of disorganizations. Keeping these facts in mind, I feel no hesitation in affirming that the proofs which I have given of these changes, whether as derived from man or from the inferior animals, cannot be resisted by any who will take pains to acquire accurate information on the subject. The examples which I myself have seen are more than I can number; and,

* See "Enquiry," pp. 277, 278.

were I to detail all the cases that have been incidentally recorded by my professional brethren, I might multiply the evidence an hundredfold. Look even at the facts stated in my "Enquiry," in the third chapter of the first part. Again, consider what is said in the second chapter of the second part. Turn, moreover, to what I have more recently published in my "Delineations," and a body of evidence will be found which, if I mistake not, must prove irresistible.

I will now direct your attention to pulmonary tubercles, which it is said receives no explanation from any of my researches; and, in fact, that all that I have advanced is a mere gratuitous hypothesis. The reviewer says, moreover, that I nowhere state that I have "traced the transformation from the vesicular or hydatidiform condition to the opaque, firm, and tubercular structure in the tissue of the lungs; and it is only by applying to these organs what he recognises in the liver, that he ascribes to this source the formation of tubercles in the human lungs."* In reference to this quotation, I might be justified in using strong language, but I will only beg you to do me the justice to consider the following facts: From the very commencement of my inquiries, I have alluded to the difficulties of acquiring any thing like accurate knowledge of the primary or elementary condition of diseased structures in the human body, for this simple reason, that we seldom or never see them till all the original characters are lost. This conviction induced me to write as follows, in page 21 of my "Illustrations:"—"When an individual affected with tubercles happens to be cut off by another disease before the tuberculous affection had run its usual course, we may sometimes be presented in the same lung with examples of all the progressive changes which I have described. Such examples, of course, cannot often occur in the human subject. It has happened to me to meet with several of this description; and I submit the following one to the reader's attentive consideration:

"A boy, about thirteen years of age, who had symptoms of pulmonary disease, was suddenly cut off by an affection of the head, and died on the 10th day of December, 1819. I examined the body on the following day. My principal attention was directed to the state of the thorax, and there I found most interesting illustrations of the descriptions given above. There were accretions nearly of the whole of the right side of the chest, but they were not so firm by any means as they are in the more advanced stage of tubercular disease.

* See Edinburgh Med. and Surg. Journal, vol. 30, p. 182.

On examining the pleura, particularly towards its upper portion, it was studded with innumerable small bodies, many of them not so large as the head of a pin. They were perfectly transparent, and glistened on the surface of the membrane. On another portion of the pleura pulmonalis, I found a tubercle, pendulous and as large as a pea, with thickened coats, and containing cheesy matter. This body is represented in plate 3d. *The transparent vesicles pervaded the substance of the lungs* as well as the membranes, but they *did not all remain in this simple or elementary form. They exhibited every gradation in the progress which has been already described.* In their first state, neither lungs nor membrane, where they occurred, were much altered; but the condition of the surrounding lung became changed with that of the tubercles themselves. *Some had lost their transparency, and were of the size of millet-seeds; others were considerably larger, and were of a firm uniform consistence;* others were less uniform both in colour and texture; some had discharged their contents, and the empty cysts appeared; others, which were consolidated, had nearly coalesced, and formed a dense, yellowish structure, quite foreign to that of the original pulmonary tissue," &c. &c.

This statement was illustrated by two plates, No. 2 and 3.

How, with this case, so circumstantially detailed, and so fully illustrated, it could have been averred that I nowhere traced the transformation of the pulmonary tubercle, I am at a loss to conceive.

I must now refer to that other part of the reviewer's statement, that it is only by applying to the lungs what I have recognised in the liver, that I assume and explain the formation of tubercles in the lungs. I am sorry to be obliged to give an unqualified contradiction to this assertion. Besides the above, I have brought forward direct proof from the inferior animals that the commencement and progress of tuberculous diseases in their lungs corresponded with the account which I have given of them in the human subject. The glandered horse affords an example of true pulmonary consumption. My examinations of that disease prove the truth of what I have asserted; and one of the engravings represents the result of one of these examinations. I, moreover, in my "Illustrations," have collected a great number of dissections from the inferior animals, as given by M. Dupuy, which amply confirm my statements. In addition to this, fig. 2 of plate 2d represents the transformations which I have described going on in the lung of a sheep, in whose liver corresponding changes were discovered. The description is most accurate.

"The figure represents a portion of the outer surface of the lung; two incisions having been made into it to show the condition of the deeper-seated changes of structure. In one, four tubercles, which had united together, were divided; and it will be observed that the appearance of the diseased mass, which is thus formed, is quite analogous to one in the cut surface of the liver. The other incision was made through a tubercle of considerable magnitude, the thickened coats of which, with the dark interior, are sufficiently apparent. In the other portions of the figure are seen a considerable number of roundish bodies, of various sizes and textures: some were firm and hard; others were less so, and almost semitransparent; while others were nearly as pellucid as that delineated in the preceding figure."

With these facts before him, and which he has actually referred to, the reviewer repeats his assertion that I have nothing but what pertains to the growth of hydatids of the liver to support my affirmations respecting changes in the lungs. He even a third time returns to this subject, and talks of the hydatids in the livers of the lower animals affording me my only pretext for predicating of tubercles in the lungs, that *their* origin and nature is similar.* His persevering in this line of argumentation, in defiance of positive proof to the contrary, can only have arisen from a desire to impugn a principle which I think I have established. It was generally believed that the part where the morbid change takes place altogether modifies and determines its nature. Thus, we are told that there is one tubercle for the lungs, another for the liver, &c. &c. A long train of observations led me to believe that this opinion is erroneous; and, though the symptoms and course of tuberculous diseases are materially influenced by the parts where they occur, that their origin is regulated by general laws connected with the essential and fundamental properties of animated beings. The conviction of this truth, which is supported by innumerable arguments, led me to declare that what was true of one organ is, *mutatis mutandis,* equally so of others. But, in making this assertion, I never dreamt of resting it on mere analogical reasoning. It was not brought forward till many dissections, and corresponding knowledge derived from the writings of my brethren, showed the same disorganization existing at the same time in the different organs of the same body. Similar experience led me to another conclusion, namely, that diversity of appear-

* See page 187 of Review.

ance did not denote diversity of origin in the disorganizations, because the appearances vary according to the progress that they have made from their primary condition. I can do no more at present than hint at these things; only further entreating my brethren to examine candidly the proofs by which they are supported.

I see full well that, notwithstanding all I have said, the reviewer, and those who think like him, will still say that my "argument is corrupted by the old fallacy, in the circumstance of regarding the hydatid as susceptible of the changes which I describe." After all that I have adduced, the amount of this objection is, that an hydatid is an hydatid, and can be nothing but an hydatid. Why, sir, the proofs that transparent cysts or vesicles, or hydatids if you will, are transmuted, and ultimately form bodies of a very different description, are so numerous and powerful, that I cannot imagine them stronger. I have witnessed this in almost every texture. Dr. Jenner established it by the most convincing evidence; and any one may satisfy himself of the fact by simply repeating the experiments which I have mentioned. But it possibly may still be said that the cysts, or vesicles, delineated by me were not hydatids. Well, be it so. In what manner is the pathological argument affected by the admission? The original state of the disorganization, and its subsequent mutations, are *proved,* and that is sufficient for my purpose.

In bringing these remarks to a conclusion, I would take the liberty of assuring those who have noticed my publications, that I am fully conscious how much the ordinarily-received professional opinions are opposed to my views. I am equally aware of the difficulty of turning the current; of inducing men to abandon long-cherished sentiments, and to adopt new ones,—to avow their errors, renounce their prejudices, and alter their whole mode of thinking and of reasoning. The force of these discouragements every one, who attempts to overthrow commonly received ideas, must experience. But there is something in the investigation of truth so sustaining and invigorating, that it keeps the inquirer steady to his purpose, in spite of all obstacles.

*Gloucester; July 7th,* 1828.

